# The genetic legacy of 50 years of desert bighorn sheep translocations

**DOI:** 10.1111/eva.12708

**Published:** 2018-10-16

**Authors:** Joshua P. Jahner, Marjorie D. Matocq, Jason L. Malaney, Mike Cox, Peregrine Wolff, Mitchell A. Gritts, Thomas L. Parchman

**Affiliations:** ^1^ Department of Biology University of Nevada Reno Nevada; ^2^ Department of Natural Resources and Environmental Science, and Program in Ecology, Evolution, and Conservation Biology University of Nevada Reno Nevada; ^3^ Department of Biology Austin Peay State University Clarksville Tennessee; ^4^ Nevada Department of Wildlife, and Wild Sheep Working Group Western Association of Fish and Wildlife Agencies Reno Nevada; ^5^ Nevada Department of Wildlife Reno Nevada; ^6^ Department of Biology, and Program in Ecology, Evolution, and Conservation Biology University of Nevada Reno Nevada

**Keywords:** genetic diversity, Great Basin Desert, Mojave Desert, Nevada, *Ovis canadensis nelsoni*, reintroduction, restoration

## Abstract

Conservation biologists have increasingly used translocations to mitigate population declines and restore locally extirpated populations. Genetic data can guide the selection of source populations for translocations and help evaluate restoration success. Bighorn sheep (*Ovis canadensis*) are a managed big game species that suffered widespread population extirpations across western North America throughout the early 1900s. Subsequent translocation programs have successfully re‐established many formally extirpated bighorn herds, but most of these programs pre‐date genetically informed management practices. The state of Nevada presents a particularly well‐documented case of decline followed by restoration of extirpated herds. Desert bighorn sheep (*O. c. nelsoni*) populations declined to less than 3,000 individuals restricted to remnant herds in the Mojave Desert and a few locations in the Great Basin Desert. Beginning in 1968, the Nevada Department of Wildlife translocated ~2,000 individuals from remnant populations to restore previously extirpated areas, possibly establishing herds with mixed ancestries. Here, we examined genetic diversity and structure among remnant herds and the genetic consequences of translocation from these herds using a genotyping‐by‐sequencing approach to genotype 17,095 loci in 303 desert bighorn sheep. We found a signal of population genetic structure among remnant Mojave Desert populations, even across geographically proximate mountain ranges. Further, we found evidence of a genetically distinct, potential relict herd from a previously hypothesized Great Basin lineage of desert bighorn sheep. The genetic structure of source herds was clearly reflected in translocated populations. In most cases, herds retained genetic evidence of multiple translocation events and subsequent admixture when founded from multiple remnant source herds. Our results add to a growing literature on how population genomic data can be used to guide and monitor restoration programs.

## 1 INTRODUCTION

Habitat fragmentation, overharvest, disease, and other human‐influenced processes are driving population declines of many species across the globe, often resulting in widespread population extirpations and putative extinctions (Cameron et al., 2011; Forister, Jahner, Casner, Wilson, & Shapiro, 2011; Lips et al., 2006; Woinarski, Burbidge, & Harrison, 2015). Translocations, where individuals from remnant populations are reintroduced into previously inhabited regions, are among the most commonly used strategies in restoration (Seddon, Griffiths, Soorae, & Armstrong, 2014). However, plant and animal translocations are financially burdensome (Weise, Stratford, & van Vuuren, 2014), vary greatly in success (Dalrymple, Banks, Stewart, & Pullin, 2012; Fischer & Lindenmayer, 2000; Godefroid et al., 2011; Griffith, Scott, Carpenter, & Reed, 1989), occasionally result in the introduction of foreign genetic ancestry (Hedrick, 2009), and can lead to the spread of disease into naïve populations (Kock, Woodford, & Rossiter, 2010). Nonetheless, there are many notable examples where translocations have restored populations (reviewed by Seddon & Armstrong, 2016). The successful reintroduction of gray wolves (*Canis lupus* L.) into the Greater Yellowstone Ecosystem led to the re‐establishment of ecosystem processes (Ripple & Beschta, 2012), while the creation of offshore populations of New Zealand's South Island saddleback (*Philesturnus carunculatus* Gmelin, 1789) prevented near‐certain extinction in response to invasive rodents (Hooson & Jamieson, 2003). These and other examples prompted the International Union for Conservation of Nature to recently update guidelines and strategies for reintroductions of species (IUCN/SSC, 2013) and promote thoughtful translocations as effective tools for maintaining and promoting biodiversity.

Translocation activities can have considerable impacts on the genetics of natural populations, including reduction in genetic variation, erosion of local adaptation, and changes to preexisting landscape genetic structure (Laikre, Schwartz, Waples, & Ryman, 2010). The preservation and augmentation of genetic variation have been principal considerations for most translocation efforts (Allendorf, Luikart, & Aitken, 2013; Biebach, Leigh, Sluzek, & Keller, 2016; IUCN/SSC, 2013; Keller, Biebach, Ewing, & Hoeck, 2012; McKay, Christian, Harrison, & Rice, 2005; Menges, 2008; Neale, 2012; Weeks et al., 2011). However, the pool of genetic variation available for reintroductions is often limited due to declines in size and connectivity of potential source populations. In extreme instances, a single small population might be the only source available for translocations, as was the case for alpine ibex (*Capra ibex* L.; Stüwe & Nievergelt, 1991), Laysan teal (*Anas laysanensis* Rothschild, 1892; Reynolds, Seavy, Vekasy, Klavitter, & Laniawe, 2008), and Mauritius kestrels (*Falco punctatus* Temminck, 1821; Jones, Heck, Lewis, Mungroo, & Cade, 1991). Even with a diverse pool of founders, many factors can result in post‐translocation loss of genetic diversity. The immediate loss of genetic diversity can be mitigated by reintroducing a large number of genetically diverse founders (Allendorf et al., 2013; Biebach et al., 2016), thus maximizing genetic diversity and providing a demographic buffer during early establishment phases (Biebach & Keller, 2012; Singer, Papouchis, & Symonds, 2000; Tracy, Wallis, Efford, & Jamieson, 2011). Once established, post‐translocation loss of genetic diversity may be slowed by allowing for gene flow among reintroduced, and possibly, native populations (Allendorf et al., 2013). However, translocations involving organisms with strong site fidelity pose unique difficulties, as these populations are naturally prone to the erosion of genetic diversity when physically isolated from other populations (Segelbacher, Höglund, & Storch, 2003; Westemeier et al., 1998).

Bighorn sheep (*Ovis canadensis* Shaw, 1804) are an iconic western North American species managed range‐wide for conservation and hunting. Historically, bighorn sheep occupied most of the rugged, high‐elevation habitats found from northern Mexico to southwestern Canada (Buechner, 1960; Valdez & Krausman, 1999), with genetically and morphologically distinct evolutionary lineages occupying separate ecological bioregions (Buchalski et al., 2016; Cowan, 1940; Malaney et al., 2015; Wehausen & Ramey, 1993, 2000 ). They are typically found in small bands of individuals that are highly faithful to natal home ranges (especially ewes), though some populations are connected via metapopulation dynamics (Bleich, Wehausen, & Holl, 1990; Bleich, Wehausen, Ramey, & Rechel, 1996; DeCesare & Pletscher, 2006; Festa‐Bianchet, 1986; Geist, 1971). In particular, rams are known for embarking on long distance, temporary movements (i.e., walkabouts) (Geist, 1971), which may contribute to gene flow and maintain genetic diversity in otherwise fragmented herds. Within herds, a subset of dominant rams typically accounts for the majority of mating events, often resulting in reproductive skew (Coltman, Festa‐Bianchet, Jorgenson, & Strobeck, 2002; Hogg & Forbes, 1997; Martin, Festa‐Bianchet, Coltman, & Pelletier, 2016; Pelletier, Hogg, & Festa‐Bianchet, 2006), the loss of genetic variation through drift, and the expectation of genetic structure across fragmented landscapes.

The most widely cited estimate of the historical number of bighorn sheep in western North America before extensive European settlement is ~1.5–2 million individuals (Seton, 1929). Dramatic declines occurred throughout the late 1800s and early 1900s, largely due to unregulated hunting, overgrazing, and susceptibility to diseases often transmitted from domestic sheep (*Ovis aries* L.) and goats (*Capra hircus* L.) (Buechner, 1960; Cassirer et al., 2018; Valdez & Krausman, 1999). Declines ultimately resulted in the extinction of at least one taxonomically contentious evolutionary lineage (Badlands bighorn sheep; *O. c. auduboni* Merriam, 1901) and perhaps other unrecognized lineages (Malaney et al., 2015). In the United States, less than 20,000 individuals remained by 1960 (Buechner, 1960), with most individuals found in small, isolated herds scattered throughout the remaining portion of the range. Several genetic consequences likely stemmed from these declines, including increased isolation of fragmented populations and reduced genetic diversity within herds.

**Figure 1:**
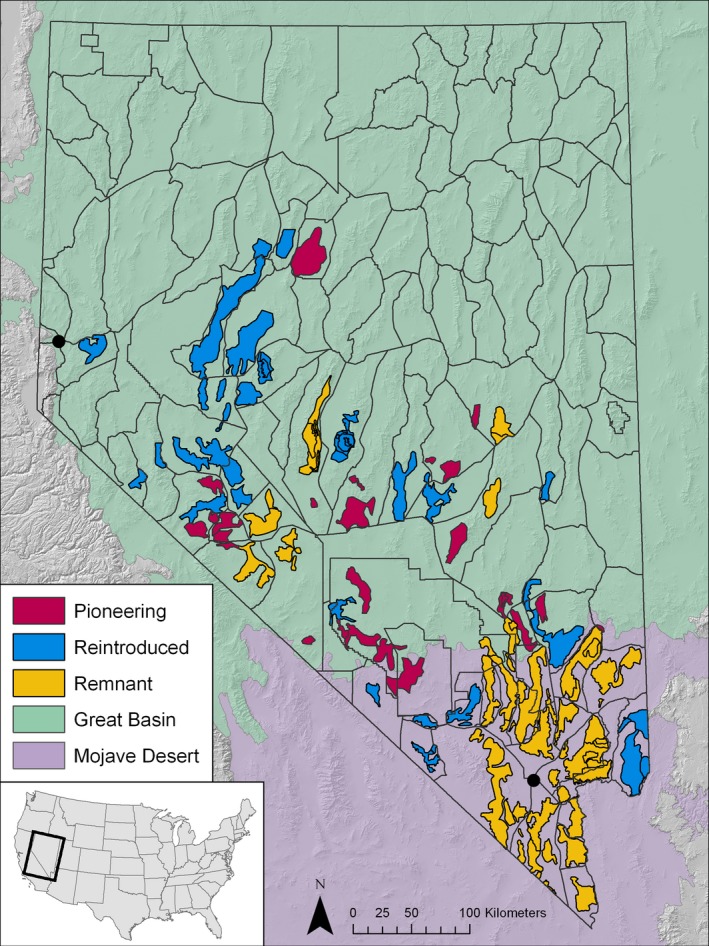
The distribution of desert bighorn sheep (*Ovis canadensis nelsoni*) in Nevada. Transparent polygons correspond to Nevada Department of Wildlife hunt units. Remnant populations (yellow) had individuals before translocations began in 1968, reintroduced populations (blue) were established via translocations after 1968, and pioneering populations (red) were naturally founded (by both ewes and rams) without human assistance after 1968. See Table S1 for the full translocation history of desert bighorn sheep in Nevada. Black circles represent the location of the two major cities in Nevada: Reno in the northwest and Las Vegas in the south. The geographic bounds of the Mojave and Great Basin Deserts (purple and green, respectively) were taken from the United States Environmental Protection Agency level III ecoregions (Omernik & Griffith, 2014)

Bighorn sheep once occupied most of Nevada's several hundred mountain ranges (Buechner, 1960; Muir, 1894). By 1960, desert bighorn sheep (*Ovis canadensis nelsoni* Merriam, 1897) populations were found primarily in the Mojave Desert, although a handful of remnant herds persisted in the Great Basin Desert (Figure 1; Buechner, 1960; Wakeling, 2015). Today, desert bighorn sheep occupy sky island mountain habitat separated by unoccupied desert valleys (Figure 1; McQuivey, 1978), though some populations are interconnected via occasional dispersal events, as seen in other high‐elevation desert mammals (Andreasen, Stewart, Longland, Beckmann, & Forister, 2012; Floyd, van Vuren, & May, 2005; Riddle, Jezkova, Hornsby, & Matocq, 2014). However, population declines and extirpations likely increased the physical and genetic isolation of remnant herds. A number of anthropogenic barriers were developed in the early to mid‐1900s that also obstructed movement among southern remnant herds (McQuivey, 1978), including the sprawling Las Vegas metropolitan area and several major highways (see Figure 1). These barriers likely further reduced the already limited gene flow among remnant herds, perhaps increasing genetic differentiation (e.g., Epps et al., 2005; Buchalski et al., 2015; but see Epps, Crowhurst, & Nickerson, 2018).

In an effort to repopulate previously occupied mountain ranges, augment genetic variation within isolated remnant herds, and increase connectivity among populations, state and federal agencies developed management programs that conducted several hundred translocations throughout the late 1900s (Wild Sheep Working Group, 2015). As part of this enterprise, the Nevada Department of Wildlife (NDOW) undertook an extensive series of translocations that have spanned five decades (1968‐present), raising the statewide population estimate from less than 3,000 individuals in 1960 to at least 12,000 individuals in 2018 (NDOW, 2001, NDOW unpublished data). NDOW's translocation strategy was guided by Cowan (1940) and Hall's (1946) hypothesis of the historic distribution of bighorn sheep: Desert bighorn sheep (*O. c. nelsoni*) were translocated into the southern two thirds of the state (typically from remnant Mojave sources), California bighorn sheep (*O. c. californiana* Douglas, 1829) into the northwest, and Rocky Mountain bighorn sheep (*O. c. canadensis* Shaw, 1804) into the northeast (Figure 1; Table S1; Malaney et al., 2015; Wakeling, 2015; Wild Sheep Working Group, 2015). It is important to note that Wehausen and Ramey (1993, 2000 ) subsequently disputed Cowan (1940) and Hall's (1946) hypothesis and instead suggested that desert bighorn sheep historically occupied all of Nevada, with a small‐horned Great Basin lineage in the north and a large‐horned Mojave lineage in the south (see Figure 1). Thus, there is uncertainty about the extent to which translocated herds may have been locally adapted to different environmental conditions, as well as the consequences of translocation history for population genetic diversity and structure across the landscape.

Here, we used genotyping‐by‐sequencing (GBS) to generate population genomic data for desert bighorn sheep sampled from the Great Basin and Mojave Deserts to explore the population genetic context and consequences of these translocations. Our goals were to: (a) characterize genetic diversity and differentiation among herds, especially among Mojave source herds; (b) evaluate patterns of admixture in reintroduced herds in light of translocation histories; and (c) quantify the degree of genetic diversity within translocated herds composed of either single or mixed origins relative to source populations. Our results provide a reference for continued translocation decisions and a baseline for understanding how past and future population responses might relate to genetic variation within and among herds. More broadly, our study illustrates how high‐throughput sequencing approaches can be used to illustrate the population genetic context and consequences of translocation activities spanning several decades.

## 2 MATERIALS AND METHODS

### 2.1 Sample DNA collection

We obtained muscle, liver, or blood samples from a hunter harvest return program and a long‐term herd‐monitoring program (NDOW). We maximized spatial coverage of samples to represent as many extant herds as possible, but actively monitored populations or those with higher harvest rates were disproportionally represented. DNA was extracted using Qiagen DNeasy Blood and Tissue Kits and quantified via spectrophotometry with a QIAxpert device (Qiagen Inc., Valencia, CA).

### 2.2 Genotyping‐by‐sequencing

Reduced‐representation libraries for Illumina sequencing were prepared using a GBS protocol (Parchman et al., 2012) analogous to ddRADseq (Peterson, Weber, Kay, Fisher, & Hoekstra, 2012). First, extracted DNA was digested using two restriction enzymes with six base pair (bp) recognition sites (*Eco*RI and *Mse*I). Next, an Illumina adaptor, an 8–10 bp barcode identifier (unique to each individual sheep), and matching sticky ends were attached to the *Eco*RI cut sites, while only an Illumina sequencing adaptor and the matching sticky ends were attached to the *Mse*I cut sites. Fragments were amplified using PCR, and the products for each individual were randomized into three separate pools. To further reduce complexity, we size selected for fragments ranging from 350 to 450 bp in size using a Pippin Prep device (Sage Science, Beverly, MA). Further details on the full library preparation protocol are available at Dryad (https://doi.org/10.5061/dryad.25f502n). Each of the three pools was sequenced on four lanes of an Illumina HiSeq 2500 at the University of Wisconsin‐Madison Biotechnology Center.

Raw sequencing data were filtered for contaminant DNA (e.g., PhiX, *E. coli*), low‐quality reads, and Illumina adaptors using bowtie2_db (Langmead & Salzberg, 2012) and a suite of bash and Perl scripts designed for this purpose (https://github.com/ncgr/tapioca). A custom Perl script was used to filter reads containing Illumina adaptor fragments, correct single or double bp errors in barcode regions (all barcodes differ by at least 3 bp), and parse reads into separate files for each individual (individual fastq files are available at Dryad; https://doi.org/10.5061/dryad.25f502n). Individuals represented by abnormally low numbers of reads were discarded. Reads from the remaining individuals were aligned to the domestic sheep genome (*O. aries* v4.0; GCF_000298735.2; Jiang et al., 2014) using the *aln* and *samse* algorithms of bwa v0.7.8 (Burrows–Wheeler aligner; Li & Durbin, 2009), with a maximum number of mismatch bases set to four. The domestic sheep genome was used as a reference instead of the Rocky Mountain bighorn sheep genome (GCA_001039535.1; Miller, Moore, Stothard, Liao, & Coltman, 2015) because a higher percentage of reads aligned to the domestic sheep genome in exploratory analyses. The resulting individual sequence alignment map (SAM) files were converted to binary alignment map (BAM) files using SAMtools v1.3 (Li et al., 2009).

Variant sites (i.e., single nucleotide polymorphisms; SNPs) were identified, and genotype likelihoods were calculated using SAMtools v1.3 and BCFtools v1.3 (Li et al., 2009). By using genotype likelihoods rather than calling genotypes categorically, these estimates reflect genotype uncertainty arising from variation in sequencing depth across individuals and loci (e.g., Nielsen, Paul, Albrechtsen, & Song, 2011). We set a minimum base quality of 20, a minimum mapping quality of 20, and required variants to have a minimum site quality of 20 and minimum genotype quality of 10. Final quality filtering of variant sites was performed with VCFtools v0.1.14 (Danecek et al., 2011), and only biallelic SNPs were retained. Loci were only included in the final dataset if their minor allele frequency (maf) was greater than 0.05 and if at least 60% of individuals had at least one read present at the locus. Additionally, individual sheep were removed if they had >50% missing data. Finally, all loci with a mean individual coverage greater than 10X were removed in an effort to filter loci that could represent mis‐assembly of paralogous regions. Considering our sequencing data represent low to medium levels of coverage, we used probabilistic methods to infer genotype probabilities while accounting for stochastic variation among loci and individuals in coverage and quality (see below).

### 2.3 Population genetic analyses

Nevada Department of Wildlife hunt management units were used to define populations for all genetic analyses. Hunt units are regions that typically encompass a major geographic feature, such as a mountain range, and often contain a single bighorn sheep population (see Figure 1). Population genetic analyses were conducted on the full quality‐filtered dataset of individuals (*N* = 303), as well as a subset of four remnant source populations that have never received translocations (*N* = 55). The four source populations included three remnant Mojave herds (unit 267/268, Black/Muddy Mountains; 269, River Mountains; and 271, Mormon Mountains) and a suspected remnant Great Basin herd on Lone Mountain (unit 212). Individuals from the Black and Muddy Mountains historically moved freely between these two contiguous mountain ranges and are considered a single population (McQuivey, 1976b, 1976c), which we hereafter refer to as the Muddy Mountains population for simplicity. Each of the remnant source populations is thought to have been demographically stable throughout the mid‐1900s (McQuivey & Leslie, 1976; McQuivey, 1976a, 1976c, 1979), though the Mormon Mountains suffered a single, rapid die‐off (>50% individual mortality) following a population expansion in the early 1980s (McQuivey, 1982). Individuals from source herds have been used to re‐establish more than half of the desert bighorn sheep herds in Nevada, as well as founding other populations in Colorado, Texas, and Utah (Wakeling, 2015; Wild Sheep Working Group, 2015).

We used a hierarchical Bayesian model (entropy; Gompert et al., 2014) that is based on the correlated allele frequency model of structure (Falush, Stephens, & Pritchard, 2003; Pritchard, Stephens, & Donnelly, 2000) to infer the number of ancestral genetic clusters (*k*), estimate ancestry coefficients for each individual, and estimate genotype probabilities for each individual at each locus. Importantly, this model utilizes a population allele frequency prior and incorporates information about genotype uncertainty arising from variation in sequencing coverage, sequence error, and alignment error during parameter estimation (Gompert et al., 2014). Numerous authors have convincingly argued that approaches that incorporate genotype uncertainty into population genetic parameter estimation are more appropriate for high‐throughput sequencing datasets including large numbers of individuals with low‐ to medium‐coverage data (e.g., Nielsen et al., 2011; Nielsen, Korneliussen, Albrechtsen, Li, & Wang, 2012; Buerkle & Gompert, 2013; Fumagalli et al., 2013). entropy has been recently employed with similar reduced‐representation sequencing data both for ancestry estimation and to infer genotype probabilities while incorporating uncertainty (e.g., Gompert et al., 2014; Lindtke, Gompert, Lexer, & Buerkle, 2014; Mandeville, Parchman, McDonald, & Buerkle, 2015; Parchman, Buerkle, Soria‐Carrasco, & Benkman, 2016; Riesch et al., 2017).

In order to determine the most probable *k* in our dataset, we conducted five replicate analyses of entropy from *k = *2 to *k = *7 for the full dataset and *k* = 2 to *k* = 4 for the remnant source dataset. The fit of each model was evaluated using a deviance information criterion (DIC), with smaller DIC values consistent with better model fit. Analyses for *k* > 7 in the full dataset and *k* > 4 in the source dataset failed to consistently converge and had high DIC values, so they were not considered further. To provide MCMC sampling with a starting point and to facilitate chain convergence, we conducted principal component analysis (PCA) on a covariance matrix of the genotype likelihoods and used *k*‐means clustering based on principal components (PCs) to cluster individuals. Starting membership probabilities to the clusters were then calculated for each individual with linear discriminant analyses (LDA) on all PCs. Both *k*‐means clustering and LDA were performed using the MASS package (Venables & Ripley, 2002) in R v3.3.3 (R Core Team, 2017). entropy analyses were based on 100,000 MCMC iterations with a burn‐in of 30,000 and thinning every tenth step. We additionally used PCA to summarize genetic variation among individuals and identify genetic structure among herds. The genotype covariance matrix from genotype probabilities generated with entropy was used as the input for PCA, which was performed using the *prcomp* function in R. The matrix of genotype probabilities is available at Dryad (https://doi.org/10.5061/dryad.25f502n).

To quantify differentiation among hunt units, Hudson's *F*_ST_ (Hudson, Slatkin, & Maddison, 1992) was calculated based on allele frequencies for each pairwise combination of hunt units with at least five genotyped individuals. To determine the relationship between pairwise *F*_ST_ and geographic distance among herds, two multiple regressions on distance matrices (MRM; Lichstein, 2007) were conducted using the *ecodist* package (Goslee & Urban, 2007) in R. A model was implemented for all populations with at least five genotyped individuals (*N* = 28), as well as for a subset of populations that have never received translocated individuals (*N* = 7; see Table 1). Haversine geographic distances were calculated using the *fossil* package (Vavrek, 2011) in R based on the midpoints of latitude and longitude for each hunt unit (Table S2).

**Table 1:** Genetic diversity of desert bighorn sheep (*Ovis canadensis nelsoni*) herds in the Great Basin (GB) and Mojave (M) Deserts are shown for hunt units with at least five genotyped individuals (*N*). The historic status and number of translocation source units are listed for each hunt unit (see Table S1 for additional details). Mean expected heterozygosity (*H*_E_), mean observed heterozygosity (*H*_O_), and heterozygote deficiency (*F*_IS_) are shown for each hunt unit

Unit	Desert	*N*	Status	*N* sources	*H*_E_	*H*_O_	*F*_IS_
131	GB	9	Remnant	2	0.190	0.167	0.118
132	GB	6	Remnant	4	0.180	0.143	0.204
161	GB	8	Reintroduced	1	0.187	0.149	0.202
173	GB	5	Remnant	2	0.192	0.178	0.072
181	GB	8	Reintroduced	1	0.189	0.160	0.152
184	GB	6	Reintroduced	1	0.184	0.152	0.176
202	GB	6	Reintroduced	6	0.190	0.165	0.134
205	GB	7	Reintroduced	4	0.191	0.157	0.180
211	GB	8	Remnant	1	0.190	0.159	0.163
212	GB	18	Remnant	0	0.178	0.157	0.122
213	GB	7	Remnant	1	0.182	0.133	0.271
223	GB	8	Reintroduced	1	0.186	0.165	0.112
241	GB/M	9	Reintroduced	4	0.196	0.176	0.105
243	M	7	Remnant	3	0.196	0.166	0.151
244	M	8	Remnant	0	0.183	0.161	0.116
245	GB	6	Remnant	1	0.187	0.173	0.074
253	M	16	Reintroduced	2	0.186	0.147	0.210
254	M	5	Reintroduced	2	0.186	0.159	0.145
261	M	5	Reintroduced	1	0.187	0.170	0.094
262	M	16	Remnant	0	0.186	0.142	0.234
263	M	16	Remnant	2	0.192	0.161	0.162
264	M	5	Remnant	1	0.185	0.141	0.238
266	M	13	Remnant	0	0.196	0.152	0.223
268	M	17	Remnant	0	0.182	0.132	0.273
269	M	10	Remnant	0	0.191	0.154	0.195
271	M	10	Remnant	0	0.177	0.154	0.131
272	M	6	Reintroduced	3	0.192	0.184	0.044
NTTR	GB/M	19	Reintroduced	2	0.201	0.190	0.054

Finally, we quantified levels of genetic diversity within each hunt unit with at least five genotyped individuals by calculating mean expected and observed heterozygosity (*H*_E_ and *H*_O_) from genotype probabilities. Mean *H*_E_ was calculated based on allele frequencies using expectations from Hardy–Weinberg equilibrium. In contrast, mean *H*_O_ was calculated directly from the genotype probabilities, where an individual was scored as heterozygous at a given locus if the genotype probability ranged between 0.9 and 1.1 (genotype probabilities for perfectly known, heterozygous loci are equal to one). Heterozygote deficiency (*F*_IS_) was calculated as *F*_IS_ =1 ‐ (*H*_O_/*H*_E_), with positive values consistent with lower than expected heterozygosity (Keller & Waller, 2002). A paired *t* test was implemented in R to assess whether *H*_O_ was significantly lower than *H*_E_ across hunt units. An additional *t* test was implemented to determine whether either *H*_E_ or *H*_O_ differed between remnant and reintroduced hunt units. Finally, linear regressions were used to assess whether number of translocation sources was positively associated with either *H*_E_ or *H*_O_.

## 3 RESULTS

Following initial filtering and the removal of individuals for which few reads were generated, we retained a dataset of 337 individuals with a mean of 2,607,602 reads per individual (sd =1,012,553 reads per individual). Initially, 1,188,258 variant sites were identified, of which 17,922 loci remained after 1) retaining only one variant site per contig, 2) keeping only biallelic SNPs, 3) removing sites with maf <0.05, and 4) excluding sites where more than 40% of individuals did not have at least one read sequenced. In the final round of filtering, individuals with more than 50% missing data were removed (*N* = 34) and loci with a mean coverage greater than ten were removed (*N* = 827; ~4.6% of total loci). This resulted in a final dataset of 303 individuals and 17,095 SNPs, with a mean coverage of 4.34X per locus per individual.

### 3.1 Genetic structure among the remnant, source herds

**Figure 2:**
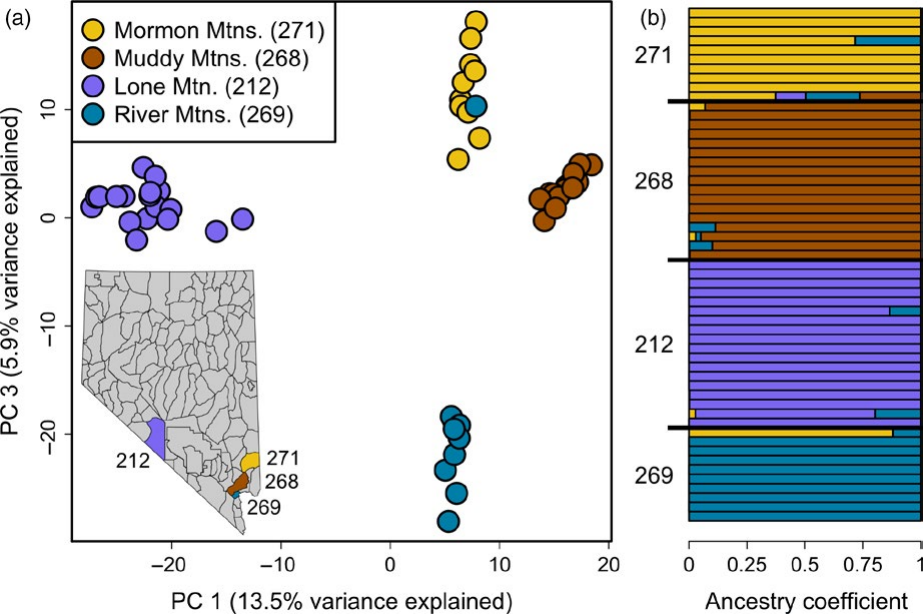
Genetic structure of four remnant source herds of desert bighorn sheep (*Ovis canadensis nelsoni*; units 212, 268, 269, and 271) based on 17,095 SNPs. (a) The first and third principal components (PCs) from PCA are plotted for every individual from the source herds. (b) An ancestry coefficient was estimated for every individual for each of four genetic ancestries (*k*) with entropy (Gompert et al., 2014)

We used a hierarchical Bayesian model (entropy) to investigate potential fine‐scale genetic structure among four source herds (units 212, 268, 269, 271) that have never received translocated individuals (Table S1). Based on DIC, the model with four genetic clusters (*k*) was better supported than models with *k* = 2 or *k* = 3 (Table S3). The four genetic clusters corresponded almost entirely to the four source herds, though an individual with high assignment to the Mormon Mountains ancestry was found in the River Mountains (Figure 2). While most other individuals had nearly 100% ancestry associated with the hunt unit they were found in, a handful of individuals showed evidence for mixed ancestry (Figure 2). Finally, one individual from the Mormon Mountains had moderate ancestry estimates from all four genetic clusters (bottom bar in Figure 2b), but this is likely a result of poor parameter estimation rather than a true representation of an individual with all four ancestries (see results from full entropy model below).

PCA based on both raw genotype likelihoods and genotype probabilities from entropy revealed patterns of population structure consistent with ancestry estimates from entropy. Specifically, the first PC explained 13.5% of the variation in the genotype probability matrix and separated individuals from the Great Basin source population (unit 212) from the three Mojave source populations (units 268, 269, and 271; Figure 2). Additionally, PC 3 explained 5.9% of the variance and clearly delineated the three Mojave source populations from one another (Figure 2). The sole individual identified by entropy as being found in the “wrong” hunt unit given its genetic ancestry was also identified in the PCA (Figure 2). Although Bayesian clustering and PCAs provide clear evidence for population structuring among these four source herds, genomewide levels of genetic divergence between these herds were not pronounced. The Great Basin remnant source population (Lone Mountain; unit 212) had the highest pairwise *F*_ST_ estimates (range =0.075 – 0.082), while the three Mojave hunt units were less differentiated from one another (range =0.063 – 0.067; Table 2).

**Table 2:** Pairwise *F*_ST_ (Hudson et al., 1992) among four remnant source herds of desert bighorn sheep (*Ovis canadensis nelsoni*)

Hunt unit	(212)	(268)	(269)	(271)
Lone Mt. (212)	–			
Muddy Mtns. (268)	0.082	–		
River Mtns. (269)	0.075	0.067	–	
Mormon Mtns. (271)	0.076	0.064	0.063	–

### 3.2 Genetic consequences of translocations

**Figure 3:**
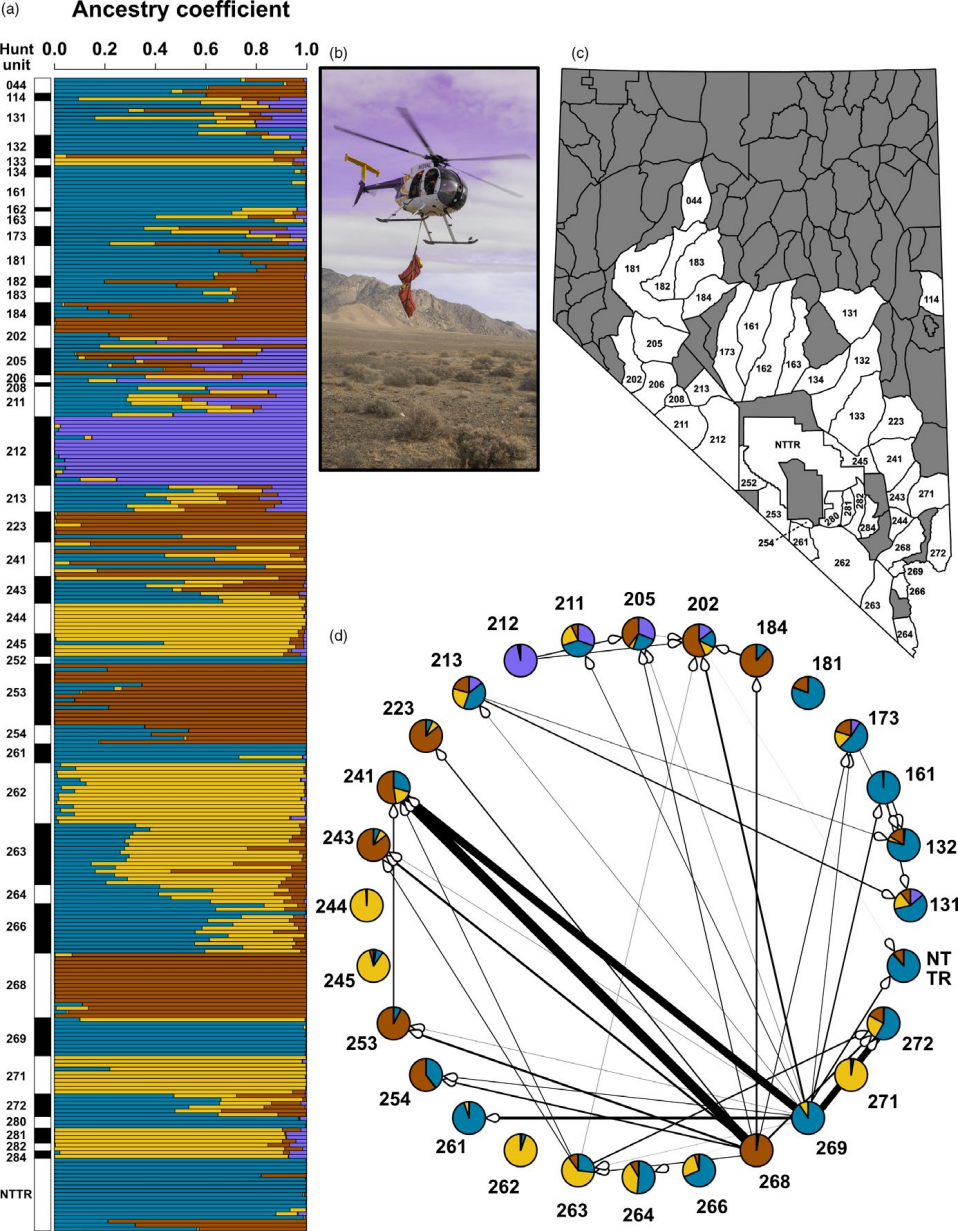
Genetic structure of remnant and reintroduced herds of desert bighorn sheep (*Ovis canadensis nelsoni*) based on 17,095 SNPs. (a) For each individual, an ancestry coefficient was estimated for each of four genetic ancestries (*k*) with entropy (Gompert et al., 2014). (b) Two desert bighorn sheep ewes from Lone Mountain (unit 212) were captured as part of a long‐term herd‐monitoring program (photograph by Robert D. Moore). (c) The distribution of hunt units sampled throughout Nevada. The Nevada Test and Training Range (NTTR) is a large government installation operated by the Department of Defense where the U.S. government tests military weapons, including nuclear weapons, prior to 1993. (d) A network illustrates the number of desert bighorn sheep that were translocated among hunt units from 1968‐present, with the size of the lines proportional to the number of translocated individuals. On each network edge, a white arrowhead denotes the hunt unit where individuals were translocated to. Pie charts display each hunt unit's mean ancestry coefficients for each of four genetic ancestries (*k*), as estimated with entropy (see panel [a] for individual ancestry coefficients). Hunt units were only included in this panel if at least five individuals were genotyped (see Table S1 for a full translocation history of all hunt units)

Comparison of the entropy models using the entire dataset of 303 individuals suggested that models from *k* = 2 to *k* = 6 had roughly equivalent support (Table S4). Qualitatively, these models were complementary to one another, consistent with the presence of hierarchical genetic structure. *k* = 6 had the lowest mean DIC and *k* = 4 had the highest mean DIC, but the magnitude of difference between all models was fairly low (Table S4). Therefore, we focus on the results from *k* = 4 because they are consistent with the results from the remnant source population analyses (Figure 2); however, we summarize entropy models for other values of *k* when appropriate (see Figs. S1, S2, S3, and S4). As expected, the four genetic clusters found in the entropy model with *k* = 4 correspond to individuals associated with the four source herds, Lone Mountain (unit 212, purple), the Muddy Mountains (268, brown), the River Mountains (269, blue), and the Mormon Mountains (271, yellow; Figure 3).

Hunt units that received translocations from multiple source herds typically had mixed ancestry reflecting both the source populations and translocation effort (Figure 3a,d). Results from the full PCA were broadly consistent with results from entropy and grouped individuals from translocated hunt units with their respective source herds (Fig. S5). For example, the Specter Range herd (unit 254) was founded by individuals from the Muddy Mountains (unit 268; brown ancestry) and River Mountains (units 268; blue ancestry) and had high ancestry coefficients for both the blue and brown genetic clusters (Figure 3). Similarly, unit 205 had roughly equivalent ancestry coefficients for the purple, brown, and blue genetic clusters, and this pattern matches a translocation history of receiving individuals from Lone Mountain (unit 212; purple ancestry), the River Mountains, and the Muddy Mountains (Figure 3). For a more thorough description of the distribution of ancestry coefficients within and among source and re‐established hunt units, see the Supplementary Results.

Across all pairwise combinations of hunt units with at least five genotyped individuals, the average pairwise *F*_ST_ was 0.054 (*N* = 378; sd =0.013; Fig. S6). *F*_ST_ values among the four remnant source populations were elevated relative to the full distribution of pairwise comparisons (Fig. S6). The minimum pairwise *F*_ST_ occurred between hunt units 253 and 268 (*F*_ST_ =0.016), while the maximum *F*_ST_ was found between hunt units 212 and 184 (*F*_ST_ =0.088; Fig. S6). The Lone Mountain herd (unit 212) had consistently elevated pairwise *F*_ST_ estimates, with all but two comparisons having *F*_ST_>0.065 (212 vs. 205 *F*_ST_ =0.052; 212 vs. 211 *F*_ST_ =0.053; Fig. S6). While the positive relationship between pairwise *F*_ST_ and geographic distance was not significant for the subset of populations (*N* = 7) that had never received translocated individuals, the model did explain over 40% of the variation in the data (MRM *R* ^2^ = 0.418; *p* = 0.074; Fig. S7a). Across all populations, there was no relationship detected between pairwise *F*_ST_ and geographic distance (MRM *R* ^2^ <0.001; *p* = 0.720; Fig. S7b), as expected based on translocation history and resulting admixture (Figure 3d).

### 3.3 Determinants of genetic diversity

Population‐level estimates of genetic diversity were calculated for hunt units with at least five genotyped individuals. Based on expectations from Hardy–Weinberg equilibrium, mean values of *H*_E_ ranged from 0.177 (unit 271; Mormon Mountains) to 0.201 (Nevada Test and Training Range [NTTR]; Table 1). Values of *H*_O_ were significantly lower than *H*_E_ (paired t_27_ = 13.6; *p* < 0.001) and ranged from 0.133 (unit 213; Monte Cristo Range) to 0.190 (NTTR; Table 1). Of the four source populations, the Muddy Mountains had lower genetic diversity (unit 268, *H*_O_ =0.136) than units 212, 269, and 271, which all had roughly equivalent mean *H*_O_ estimates (*H*_O_ =0.154 – 0.157). Relative to remnant populations, reintroduced populations did not have reduced levels of *H*_E_ (remnant *H*_E_ =0.187; reintroduced *H*_E_ =0.190; t_25.671_ = 1.419; *p* = 0.168) or *H*_O_ (remnant *H*_O_ =0.155; reintroduced *H*_O_ =0.164; t_23.889_ = 1.871; *p* = 0.074). Finally, the number of translocation sources revealed a marginally positive (but statistically insignificant) association with both *H*_E_ (*F*_1,26_ = 4.12; *R* ^2^ = 0.137; *p* = 0.053; Fig. S8a) and *H*_O_ (*F*_1,26_ = 3.44; *R* ^2^ = 0.117; *p* = 0.075; Fig. S8b).

## 4 DISCUSSION

### 4.1 Fine‐scale genetic structure among remnant Mojave populations

Our results illustrate fine‐scale population genetic structure among remnant populations of desert bighorn sheep in the northern Mojave Desert (Figures 2 and 3), although levels of differentiation among source populations were generally low (*F*_ST_ range: 0.063–0.082; Table 2). Two geographically proximate Mojave source populations (Muddy and River Mountains) were clearly differentiated from both one another and other remnant Mojave populations (Table 2; Figures 2, 3, and S6), which instead appear to be associated with a historically connected metapopulation that now encircles Las Vegas (Figure 3; see Supplemental Results). Our results suggest evidence of population genetic structure across finer spatial scales than most past genetic studies of North American wild sheep, which detected genetic structure across various spatial scales using a broad range of molecular markers (e.g., Ramey, 1995; Luikart & Allendorf, 1996; Fitzsimmons, Buskirk, & Smith, 1997; Boyce, Ramey, Rodwell, Rubin, & Singer, 1999; Gutiérrez‐Espeleta, Kalinowski, Boyce, & Hedrick, 2000; Worley et al., 2004; Miller, Poissant, Kijas, & Coltman, 2011; Buchalski et al., 2015, 2016 ; Kardos et al., 2015; Malaney et al., 2015; Sim, Hall, Jex, Hegel, & Coltman, 2016). This increased spatial resolution could be a product of the geographic distribution of isolated mountain ranges that bighorn sheep occupy in Nevada (Figure 1), but was also likely influenced by the relatively large number of markers we employed (~17,000 SNPs) compared to most past studies.

The relatively fine geographic scale at which we detected genetic structure is likely associated with both the natural history of bighorn sheep and the recent history of human activity in this region. First, the life history of bighorn sheep predisposes populations to genetic differentiation, as individuals often exhibit high site fidelity to natal habitats with access to high‐quality forage, escape terrain, and water sources (McQuivey, 1978). Furthermore, the skewed mating ratio of bighorn sheep, where a few rams account for most of the successful mating events, (Coltman et al., 2002; Hogg & Forbes, 1997; Martin et al., 2016; Pelletier et al., 2006) can lower effective population sizes, intensify genetic drift within herds, and lead to population structure across increasingly fragmented landscapes, as seen in other organisms with polygynous mating systems (e.g., Coltman, Pilkington, & Pemberton, 2003; Bouzat & Johnson, 2004; Shafer, Côté, & Coltman, 2011; Jahner et al., 2016; Dotsev et al., 2018). Additionally, desert bighorn sheep are found in naturally fragmented mountainous habitats, with occasional movements across low‐elevation basins presumably allowing for gene flow. Such movements are only possible if the span of unsuitable habitat is narrow and if there are limited anthropogenic physical barriers to movement (e.g., large roads or fences) (Epps, Wehausen, Bleich, Torres, & Brashares, 2007). While our results support historic differentiation with some connectivity among remnant Mojave populations (Figure 3, S4, and S6), the development of several anthropogenic barriers to movement could have enhanced isolation among herds over the past century.

The potential impact of human activities on desert bighorn genetic structure is perhaps best illustrated by the two most commonly used Mojave source populations, the Muddy Mountains and River Mountains (units 268 and 269). Individuals from these herds were historically dependent on regular movements and access to the nearby Colorado River for water (the southeastern border of Nevada; Figure 1; McQuivey, 1976b; McQuivey, 1976c; McQuivey & Leslie, 1976), increasing the opportunity for gene flow between these geographically proximate populations (hunt unit midpoints separated by 31.3 km). In the 1930s, an extensive network of roads and infrastructure was developed to support the construction of the Hoover Dam on the Colorado River (completed in 1936), effectively isolating the River Mountains herd from other populations (Leslie, 1977). Subsequently, a number of artificial water sources (i.e., guzzlers) were installed in the River Mountains (Leslie & Douglas, 1979; McQuivey & Leslie, 1976), minimizing the need for individuals to travel for water. Despite past reports suggesting that individuals migrated between the Muddy and River Mountains (Denniston, 1965), our results suggest substantial gene flow has not recently occurred between these herds (Figure 2). Thus, these naturally differentiated herds could have been further isolated and subdivided by the past 80 years (~11 sheep generations) of human activity and infrastructure. It is worth noting, however, that the effectiveness of anthropogenic barriers to sheep dispersal can vary substantially over time (Epps et al., 2018), and future changes could influence the degree of differentiation between these two remnant populations.

### 4.2 Remnant Great Basin genetic ancestry

The original strategy for repopulating bighorn sheep to the Great Basin Desert was guided by a contentious taxonomic hypothesis (Cowan, 1940; Hall, 1946). Our results could lend support to Wehausen and Ramey's (1993, 2000 ) alternative hypothesis of a historically widespread desert bighorn sheep range, with cranial morphology and population substructure strongly matching ecotypic differences among western North American deserts (i.e., a small‐horned Great Basin lineage and a large‐horned Mojave lineage). The herd we sampled on Lone Mountain (unit 212) has one of the smallest mean horn sizes in Nevada (M. Cox, personal observation) and appears to be resistant to pneumonia despite the recent detection of *Mycoplasma ovipneumoniae* in the population (NDOW, 2017). This is also the most genetically differentiated population we sampled (Table 2, Figure S6) and may thus be a relict of an unrecognized Great Basin lineage of desert bighorn sheep. Nonetheless, the magnitude of differentiation between Lone Mountain and the Mojave source herds is modest (Table 2), so it is possible that the remnant Great Basin populations are simply recently differentiated herds that may be locally adapted to an ecosystem that differs dramatically from the Mojave Desert in both climate and vegetation (Beatley, 1975; Pavlik, 2008). Preliminary results suggest that a herd with remnant Great Basin ancestry may also be present in the White Mountains of California (M. R. Buchalski, personal communication), and future research should compare the isolation of these Great Basin remnant herds to the degree of differentiation found among other recognized desert bighorn sheep lineages (Mexican, Nelson's, and Peninsular desert bighorns; Buchalski et al., 2016). Such results suggest that more comprehensive geographic and genetic sampling could contribute significantly to future translocation decisions.

If remnant Great Basin herds are indeed locally adapted and independent from Mojave populations, then conservation strategies should ideally manage desert bighorn sheep as multiple evolutionary significant units (Moritz, 1999). Such a strategy could maintain the evolutionary legacy of the Lone Mountain herd as a relict of the Great Basin lineage. However, NDOW has been encouraged by some hunters to translocate rams from large‐horned Mojave populations into remnant small‐horned herds like Lone Mountain to increase mean horn size and augment trophy hunting opportunities. Such translocations could have negative consequences for the preservation of remnant Great Basin ancestry. Moreover, horn size in ungulates is influenced by both environmental and genetic factors (Monteith et al., 2018), so translocations of large‐horned rams may have limited phenotypic consequences. Sourcing Lone Mountain individuals for translocations to other Great Basin habitat could be advantageous because such individuals might be better adapted to Great Basin habitats than their Mojave counterparts (Malaney et al., 2015; Wehausen, 1991). For example, North Dakotan bighorn sheep had greater recruitment and projected population growth rates when they were sourced from environmentally similar populations (Bleich, Sargeant, & Wiedmann, 2018), lending support to recent calls for managers to consider using ecologically similar source populations for translocations (e.g., Lawrence & Kaye, 2011; Malaney et al., 2015; Biebach et al., 2016; Kronenberger et al., 2017). Although inferences regarding local adaptation across these regions are not currently available, translocation of sheep from ecologically mismatched habitats could establish new populations with maladapted individuals or introduce maladaptive genetic variation into existing populations. Although marker density and sampling effort in this study (~1 locus per 200 kb) are not suited to detecting genomic regions involved in local adaptation (see Lowry et al., 2017a, 2017b), more comprehensive individual sampling and higher marker densities should support genomic analyses for local adaptation in future work.

### 4.3 The genetic legacy of 50 years of translocations

The composition of genetic ancestry within reintroduced populations of bighorn sheep is strongly defined by the history of translocation. Individuals from herds founded by a single source had high ancestry coefficients for the corresponding source's genetic ancestry (Figure 3), suggesting that natural movements among populations after translocations were either infrequent or had a minimal impact on current genetic variation. In addition, individuals from herds established from multiple sources typically had admixed ancestry representing the corresponding multiple source populations (Figure 3), suggesting individuals from all of the distinct source herds successfully interbred. Furthermore, these multiple source translocations resulted in offspring with mosaic genomes (Figure 3) that seem to be persisting in these environments, as opposed to retaining a signature of population genetic structure in subsequent generations (e.g., Muller et al., 2018). Encouragingly, reintroduced populations did not significantly differ from remnant populations in levels of genetic diversity (both *H*_E_ and *H*_O_; see Table 1). There was marginal evidence for elevated diversity in multiple source herds relative to single source herds (Figure S8), which has also been reported for California bighorn sheep populations in Oregon (Olson, Whittaker, & Rhodes, 2013). The maintenance of genetic diversity in these reintroduced populations contrasts with studies of alpine ibex (*Capra ibex*), where serial reintroductions over the past century resulted in less diverse populations containing only a subset of the source's genetic diversity (Biebach & Keller, 2009; Grossen, Biebach, Angelone‐Alasaad, Keller, & Croll, 2018). In general, studies of genetic diversity in reintroduced populations have yielded inconsistent patterns, finding evidence for both reduced diversity (e.g., Krauss, Dixon, & Dixon, 2002; Mock, Latch, & Rhodes, 2004; Sigg, 2006) and no loss in diversity following translocation (e.g., Larson, Jameson, Bodkin, Staedler, & Bentzen, 2002; Hicks, Rachlow, Rhodes, Williams, & Waits, 2007; Williams & Scribner, 2010; Wright et al., 2014). Future studies should continue to focus on identifying potential links between demographic parameters and diversity metrics across remnant and reintroduced populations in addition to decoupling the complex history of translocations.

When developing a translocation program, managers balance short‐term and long‐term risks of population health to ensure success (Jamieson & Lacy, 2012). Translocations of bighorn sheep to maintain genetic diversity pose one such trade‐off, as translocations reduce the potential effects of inbreeding depression within a population and, at the same time, increase the potential for disease transmission and could compromise local adaptation. Across the range of bighorn sheep, populations have suffered recent die‐offs in response to pneumonia outbreaks (Besser et al., 2013; Cassirer et al., 2018), occasionally resulting in the extirpation of populations. The severe negative impacts of these disease outbreaks have led NDOW to take the aggressive approach of culling wandering rams that may infect neighboring populations. Given the potential for translocations to spread disease among populations (Cassirer et al., 2018), we recommend that translocations to augment genetic diversity in already established populations only be undertaken when signs of inbreeding depression have been directly identified within a population (e.g., lower survival for inbred female lambs; Rioux‐Paquette, Festa‐Bianchet, & Coltman, 2011). While this strategy is less than ideal for managing fragmented populations that are at elevated risk of losing genetic diversity (Frankham et al., 2017), the short‐term risks of disease transmission are currently too high to justify attempts to mitigate the more long‐term risks of reduced genetic diversity. Looking forward, future success in this system will depend on how well translocations are leveraged to maintain genetic variation and maximize population persistence while preserving the identities of multiple evolutionary ancestries.

### 4.4 Informing restoration with population genomic variation

Even prior to the advent of modern sequencing technologies, a pivotal question in restoration genetics was whether translocation programs could establish populations with sufficient levels of genetic diversity. Maintaining diversity could be important for avoiding inbreeding depression and providing sufficient genetic variation for adaptation. Although genetic diversity can be maintained by choosing a large and diverse pool of founders, genetic surveys of potential source individuals prior to translocation are rarely utilized (but see Shultz, Baker, Toonen, Harting, & Bowen, 2011). In an attempt to proceed with caution, managers have typically targeted their efforts to augment diversity in those populations that show clear evidence of loss of genetic diversity or signs of inbreeding depression (i.e., genetic rescue; Frankham, 2015; Whiteley, Fitzpatrick, Funk, & Tallmon, 2015). Genetic rescue has been effectively employed in a number of cases, including Florida panthers (*Puma concolor* (L.); Johnson et al., 2010), greater prairie chickens (*Tympanuchus cupido* (L.); Westemeier et al., 1998), and European adders (*Vipera berus* (L.); Madsen, Shine, Olsson, & Wittzell, 1999). However, the benefits of genetic rescue can be short‐lived if only a few individuals are moved, as seen in the Isle Royale population of gray wolves (Hedrick, Peterson, Vucetich, Adams, & Vucetich, 2014), and continued augmentations to sustain genetic diversity significantly increase the financial burden of translocation programs. Encouragingly, our results suggest that translocating individuals from multiple genetically differentiated, although geographically proximate, source populations was effective at maintaining genetic diversity in newly founded populations. Thus, even though genetic rescue has been demonstrated to be an effective tool for restoring genetic diversity in small populations of organisms (Frankham, 2015), including bighorn sheep (e.g., Hogg, Forbes, Steele, & Luikart, 2006; Gompert, 2012; Miller, Poissant, Hogg, & Coltman, 2012; Olson, Whittaker, & Rhodes, 2012), more widespread genetic sampling of potential source populations prior to translocation may be an effective approach.

Although several thousand translocations have been conducted over the past century without genetic monitoring (Laikre et al., 2010), much insight could be gained by analyzing the population genetic context and consequences of restoration programs. Such analyses can provide a survey of genetic variation across candidate source populations, illustrate how translocation activities alter landscape genetic variation, and provide insight into evolutionary history that may be relevant for understanding local adaptation. In general, large‐scale translocations will greatly impact the landscape genetic structure of populations relative to preexisting natural conditions. For example, a study of 72 lake trout (*Salvelinus namaycush* Walbaum 1792) populations found that natural lakes had lower genetic diversity and higher genetic differentiation than stocked lakes (Valiquette, Perrier, Thibault, & Bernatchez, 2014). Similarly, in our study, reintroduced populations lacked any signature of isolation by distance (Fig. S7b) and pairwise *F*_ST_ values among remnant source populations were elevated relative to those among re‐established populations (Fig. S6). Higher density population genetic data can also reveal previously unrecognized genetic structure (e.g., the Lone Mountain herd in our study) that could represent unique patterns of evolutionary history potentially associated with local adaptation. The preservation of locally adapted populations is a primary goal of many restoration efforts (McKay et al., 2005; Weeks et al., 2011), and while the presence of population genetic structure alone does not provide evidence for local adaptation, managers often consider the preservation of genetically differentiated populations with unique phenotypic variation. Finally, genetic data can identify natural corridors that allow for individual movements among populations that can be important for maintaining genetic diversity without human assistance (e.g., the pioneering herds in Figure 1; Epps, Wehausen, Palsbøll, & McCullough, 2010; Gilbert‐Norton, Wilson, Stevens, & Beard, 2010). As the generation of high‐throughput DNA sequencing data has become rapid and cost‐effective, the analysis of such data could increasingly be used to guide and assess the restoration of populations.

### 4.5 Conclusions

Emerging technologies that allow for increases in the extent of genomic sampling are now reshaping our understanding of the genetic consequences of conservation actions, even for organisms with complicated management histories (Allendorf, Hohenlohe, & Luikart, 2010; Shafer et al., 2015). However, relatively few studies have utilized such datasets to investigate the genetic legacy of reintroduction programs (e.g., Campbell, Kamphaus, Murdoch, & Narum, 2017; Grossen et al., 2018). By employing a GBS approach to investigate the distribution of desert bighorn sheep genetic variation across the Great Basin and northern Mojave Deserts, we uncovered an intricate genetic landscape structured by 50 years of translocation decisions. Furthermore, our results support the possible existence of a previously hypothesized Great Basin lineage of desert bighorn sheep that may require revised management consideration. The population genetic patterns identified here will serve as a baseline that can be used to inform future translocation decisions, as well as a reference to understand how future population responses to disease outbreaks, climate change, and other environmental challenges are affected by genetic diversity and variation.

## DATA ARCHIVING STATEMENT

The GBS library preparation protocol, individual fastq files, and a matrix of genotype probabilities are available from the Dryad Digital Repository: https://doi.org/10.5061/dryad.25f502n.
