# Supplementary material for: The genetic legacy of 50 years of desert bighorn sheep translocations
Source: Evol Appl. 2018 Oct 16;12(2):198–213. doi: 10.1111/eva.12708 (PMC6346675; doi:10.1111/eva.12708)
Supplement: Supplementary file 1 [file EVA-12-198-s001.docx]

**Supporting Information**

**The genetic legacy of 50 years of desert bighorn sheep translocations**

Joshua P. Jahner **|** Marjorie D. Matocq **|** Jason L. Malaney **|** Mike Cox **|** Peregrine Wolff **|** Mitchell A. Gritts **|** Thomas L. Parchman

**Supplementary Results**

As in the remnant source population entropy model (Fig. 2), individuals from Lone Mountain (unit 212; the Great Basin source) had high ancestry coefficients for the purple ancestry (Fig. 3). Individuals with moderate ancestry coefficients for the purple genetic cluster were also found in two hunt units (202 and 205) that received translocated individuals from Lone Mountain (Table S1), as well as the geographically proximate Silver Peak Range (unit 211). While individuals from the White Pine and Monte Cristo Ranges (units 131 and 213) also had individuals with moderate ancestry coefficients for the purple genetic cluster, this result disappears in entropy models with higher values of *k* (Fig. S3 and S4). Instead, models of *k* = 5 and *k* = 6 suggest the Monte Cristo Range is a Great Basin remnant herd with a potentially unique genetic ancestry (the White Pine Range received translocated individuals from the Monte Cristo Range; Fig. 3; Table S1).

The brown genetic cluster from the *k* = 4 entropy model corresponds to ancestry associated with the Muddy Mountains source herd (unit 268). All individuals from the Muddy Mountains had high ancestry coefficients for the brown genetic cluster (Fig. 3), consistent with results of the remnant source population entropy model (Fig. 2). Several other populations that received translocated individuals from the Muddy Mountains also had moderate to high ancestry coefficients for the brown genetic cluster, including units 173, 184, 205, 223, 253, 254, and 272 (Fig. 3). The Wassuk Range population (unit 202) had greater than 50% mean ancestry coefficients for the brown genetic cluster despite never receiving translocated individuals from the Muddy Mountains source (Fig. 3D). However, the Wassuk Range did receive translocated individuals from hunt unit 184, which does have moderate to high ancestry coefficients for the brown genetic cluster, presumably because hunt unit 184 received translocations directly from the Muddy Mountains (Fig. 3; Table S1).

All but one individual from the River Mountains source herd (unit 269) had high ancestry coefficients for the blue genetic cluster (Fig. 3). The sole exception was an individual with a high ancestry coefficient for the yellow genetic cluster (associated with the Mormon Mountains source; unit 271) in both the full and remnant source entropy models (Figs. 2 & 3). Individuals with moderate to high ancestry coefficients to the blue genetic cluster were found in hunt units 044, 134, 161, 163, 182, 183, 243, 261, 263, 264, 280, and the Nevada Test and Training Range (NTTR), all of which have received translocated individuals from the River Mountains (Fig. 3; Table S1). Three hunt units (132, 181, and 266) had high ancestry coefficients for the blue genetic cluster even though they never directly received individuals from the River Mountains (Fig. 3; Table S1). The presence of the River Mountains genetic ancestry in units 132 and 181 is likely attributable to translocations from other hunt units with high ancestry coefficients to the blue genetic cluster (units 161 and 134, respectively; Table S1). However, natural movement of individuals from the River Mountains is a more likely explanation for hunt unit 266, as this population has never received translocated individuals (Table S1).

The fourth genetic cluster (yellow) had high ancestry coefficients for all individuals from the Mormon Mountains source population (hunt unit 271). Individuals from several other remnant populations also had very high ancestry coefficients to the yellow genetic cluster, including units 133, 244, 245, 262, 281, 282, and 284 (Fig. 3), suggesting that the yellow genetic ancestry is associated with a more widespread, remnant Mojave metapopulation that surrounds Las Vegas. Moderate ancestry coefficients for the yellow genetic cluster were also found in several other remnant Mojave herds (e.g. units 243, 263, 264, and 266), but the historical genetic signature of these herds have been obscured by past translocations or natural movements from nearby source herds (Fig. 3). Results from the *k* = 6 entropy model suggest that herds associated with the yellow genetic cluster (*k* = 4 model) could be further subdivided, with hunt units 263, 264, and 266 found in one group and hunt units 133, 244, 245, 271, 281, 282, and 283 found in the other group (Fig. S3).

| **Table S1.** The entire history of desert bighorn sheep translocations within the state of Nevada from 1968-present (imports and exports are not included; see Wild Sheep Working Group, 2015). In total, 2,111 individuals were moved during 137 separate translocations. | | | |
| --- | --- | --- | --- |
| Year | *N* | Release site (hunt unit) | Capture site (hunt unit) |
| 1968 | 6 | Wassuk Range (202) | Corn Creek (Desert NWR) (284) |
| 1969 | 10 | Wassuk Range (202) | River Mountains (269) |
| 1970 | 5 | Wassuk Range (202) | Highland Range (263) |
| 1974 | 6 | Wassuk Range (202) | Various sites, Clark County (unknown) |
| 1975 | 8 | Stonewall Mountain (NTTR) | River Mountains (269) |
|  | 4 | Wassuk Range (202) | Corn Creek (Desert NWR) (284) |
| 1977 | 13 | Stonewall Mountain (NTTR) | River Mountains (269) |
| 1979 | 21 | Virgin Mountains (272) | River Mountains (269) |
| 1980 | 22 | Virgin Mountains (272) | River Mountains (269) |
|  | 1 | Stonewall Mountain (NTTR) | Wassuk Range (202) |
| 1981 | 20 | South Stillwater Range (182) | Black Mountains (267/268) |
| 1982 | 18 | Hot Creek Range (163) | River Mountains (269) |
|  | 22 | Mt. Jefferson (161) | River Mountains (269) |
|  | 4 | Toiyabe Range (173) | River Mountains (269) |
|  | 25 | South Stillwater Range (182) | River Mountains (269) |
| 1983 | 7 | Monte Cristo Range (213) | River Mountains (269) |
|  | 6 | Hot Creek Range (163) | River Mountains (269) |
|  | 10 | Stonewall Mountain (NTTR) | River Mountains (269) |
|  | 4 | Mt. Jefferson (161) | River Mountains (269) |
|  | 4 | Silver Peak Range (211) | River Mountains (269) |
|  | 20 | Gold Buttes (272) | River Mountains (269) |
|  | 22 | Pilot Mountains (207) | River Mountains (269) |
| 1984 | 26 | Pancake Range (134) | River Mountains (269) |
|  | 30 | East Range (044) | River Mountains (269) |
|  | 34 | South Tobin Range (045) | River Mountains (269) |
|  | 5 | South Stillwater Range (182) | River Mountains (269) |
|  | 17 | Toiyabe Range (173) | Black Mountains (267/268) |
| 1985 | 19 | South Pahroc Range (241) | Black Mountains (267/268) |
|  | 10 | Middle Stillwater Range (182) | Black Mountains (267/268) |
| 1986 | 2 | Excelsior Mountains (206) | River Mountains (269) |
|  | 14 | Excelsior Mountains (206) | Lone Mountain (212) |
|  | 15 | Wassuk Range (202) | Lone Mountain (212) |
|  | 2 | Wassuk Range (202) | River Mountains (269) |
|  | 1 | Pilot Mountain (207) | River Mountains (269) |
|  | 31 | Clan Alpine Range (183) | River Mountains (269) |
|  | 30 | Desatoya Mountains (184) | Black Mountains (267/268) |
|  | 19 | South Egan Range (221) | Black Mountains (267/268) |
|  | 17 | South Pahroc Range (241) | Black Mountains (267/268) |
|  | 11 | Middle Stillwater Range (182) | Black Mountains (267/268) |
|  | 9 | Silver Peak Range (211) | River Mountains (269) |
| 1987 | 10 | North Hiko Range (223) | Black Mountains (267/268) |
|  | 14 | North Hiko Range (223) | Black Mountains (267/268) |
|  | 14 | North Stillwater Range (182) | Black Mountains (267/268) |
|  | 14 | Gold Buttes (272) | Black Mountains (267/268) |
|  | 22 | South Hiko Range (241) | Black Mountains (267/268) |
| 1988 | 24 | Last Chance Range (261) | River Mountains (269) |
|  | 22 | North Gabbs Valley Range (205) | Lone Mountain (212) |
|  | 2 | Wassuk Range (202) | Lone Mountain (212) |
| 1989 | 1 | Wassuk Range (202) | River Mountains (269) |
|  | 2 | Excelsior Mountains (206) | River Mountains (269) |
|  | 2 | Pilot Mountains (207) | River Mountains (269) |
|  | 3 | North Gabbs Valley Range (205) | River Mountains (269) |
|  | 25 | Last Chance Range (261) | River Mountains (269) |
|  | 7 | Gold Buttes (272) | River Mountains (269) |
|  | 15 | North Stillwater Mtns (182) | Black Mountains (267/268) |
|  | 11 | Clan Alpine Mountains (183) | Black Mountains (267/268) |
| 1990 | 12 | South Gabbs Valley Range (207) | Black Mountains (267/268) |
|  | 9 | Specter Range (254) | River Mountains (269) |
|  | 10 | Specter Range (254) | Muddy Mountains (268) |
|  | 12 | Pahranagat Range (245) | Sheep Range (283/284) |
| 1991 | 3 | Pahranagat Range (245) | Sheep Range (283/284) |
|  | 20 | Bare Mountains (253) | Black Mountains (267/268) |
|  | 18 | North Tobin Range (045) | Black Mountains (267/268) |
| 1993 | 20 | South Egan Range (221) | Mormon Mountains (271) |
|  | 17 | Mid. Gabbs Valley Range (205) | Muddy Mountains (268) |
|  | 3 | Mid. Gabbs Valley Range (205) | River Mountains (269) |
|  | 20 | Specter Range (254) | Muddy Mountains (268) |
|  | 18 | Bare Mountains (253) | Muddy Mountains (268) |
|  | 21 | Pine Grove Range (204) | Mormon Mountains (271) |
|  | 25 | Spotted Range (280) | River Mountains (269) |
|  | 9 | Toiyabe Range (173) | River Mountains (269) |
| 1994 | 21 | Hot Creek Range (163) | Mormon Mountains (271) |
|  | 16 | Highland Range (263) | Muddy Mountains (268) |
|  | 3 | Highland Range (263) | River Mountains (269) |
|  | 22 | Wassuk Range (202) | River Mountains (269) |
|  | 1 | Pine Grove Range (204) | River Mountains (269) |
|  | 1 | Wassuk Range (202) | Gabbs Valley Range (205) |
| 1995 | 21 | Pine Grove Range (204) | River Mountains (269) |
|  | 5 | Specter Range (254) | River Mountains (269) |
|  | 5 | Bare Mountains (253) | River Mountains (269) |
|  | 21 | Hot Creek Range (163) | Muddy Mountains (268) |
|  | 15 | East Range (044) | Muddy Mountains (268) |
| 1996 | 5 | Middle Sheep Range (284) | East Pahranagat Range (245) |
|  | 25 | Spotted Range (280) | River Mountains (269) |
|  | 15 | Sand Springs Range (181) | Pancake Range (134) |
|  | 16 | Slate Mountain (181) | Pancake Range (134) |
|  | 1 | Newberry Mountains (264) | River Mountains (269) |
| 1997 | 19 | Delamar Mountains (241) | River Mountains (269) |
| 1998 | 21 | Gillis Range (205) | Pancake Range (134) |
|  | 10 | South Sheep Range (284) | Muddy Mountains (268) |
|  | 20 | Gold Buttes (272) | Muddy Mountains (268) |
|  | 10 | South Sheep Range (284) | Arrow Canyon Range (244) |
|  | 15 | South Sheep Range (284) | Specter Range (254) |
| 1999 | 25 | White Pine Range (131) | Monte Cristo Range (213) |
|  | 25 | Delamar Mountains (241) | River Mountains (269) |
| 2001 | 22 | Pine Grove Range (204) | Gabbs Valley Range (205) |
|  | 26 | Delamar Mountains (241) | Muddy Mountains (268) |
| 2002 | 25 | Clan Alpine Range (183) | Toquima Range (161) |
| 2003 | 15 | Delamar Mountains (241) | McCullough Range (263) |
|  | 10 | Delamar Mountains (241) | Muddy Mountains (268) |
|  | 23 | East Range (044) | Pancake Range (134) |
|  | 25 | South Tobin Range (045) | Toquima Range (161) |
| 2005 | 25 | Virgin Mountains (272) | River Mountains (269) |
|  | 4 | Grant Range (132) | Pilot Mountain (207) |
|  | 10 | Grant Range (132) | Monte Cristo Range (213) |
|  | 12 | Grant Range (132) | Toiyabe Range (173) |
| 2006 | 27 | Virgin Mountains (272) | McCullough Range (263) |
| 2007 | 22 | Grant Range (132) | Toquima Range (161) |
|  | 24 | White Pine Range (131) | Toquima Range (161) |
|  | 25 | Delamar Mountains (241) | Muddy Mountains (268) |
|  | 28 | Delamar Mountains (241) | River Mountains (269) |
|  | 28 | Wassuk Range (202) | Desatoya Range (184) |
| 2008 | 22 | South Tobin Range (045) | Toquima Range (161) |
|  | 50 | Delamar Mountains (241) | River Mountains (269) |
|  | 25 | Delamar Mountains (241) | Muddy Mountains (268) |
|  | 16 | Meadow Valley Mtns (243) | Muddy Mountains (268) |
|  | 16 | Meadow Valley Mtns (243) | McCullough Range (263) |
| 2009 | 25 | Stillwater Range (182) | Silver Peak Range (211) |
| 2010 | 5 | Meadow Valley Mtns (243) | River Mountains (269) |
|  | 11 | Delamar Mountains (241) | River Mountains (269) |
|  | 5 | Virgin Mountains (272) | River Mountains (269) |
|  | 4 | Gold Buttes (272) | River Mountains (269) |
| 2011 | 20 | Excelsior Mountains (206) | Stonewall Mountain (NTTR) |
|  | 8 | Virginia Range (195) | Stonewall Mountain (NTTR) |
|  | 17 | Gold Buttes (272) | River Mountains (269) |
|  | 8 | Delamar Mountains (241) | River Mountains (269) |
|  | 27 | Delamar Mountains (241) | Muddy Mountains (268) |
|  | 24 | Meadow Valley Mtns (243) | Muddy Mountains (268) |
|  | 25 | South Pahroc Range (241) | Bare Mountains (253) |
|  | 34 | Virginia Range (195) | Monte Cristo Range (213) |
| 2012 | 11 | Virginia Range (195) | River Mountains (269) |
|  | 7 | Excelsior Mountains (206) | River Mountains (269) |
|  | 25 | Excelsior Mountains (206) | Lone Mountain (212) |
| 2013 | 30 | Candelaria Hills (208) | Bare Mountains (253) |
|  | 20 | Excelsior Mountains (206) | Bare Mountains (253) |
| 2015 | 15 | Garfield Hills (206) | Gabbs Valley Range (205) |
| 2016 | 17 | Garfield Hills (206) | Lone Mountain (212) |

| **Table S2.** Number of sequenced individuals (*N*) and geographic centers of each hunt unit. The midpoint | | | |
| --- | --- | --- | --- |
| Hunt unit | *N* | Latitude | Longitude |
| 044 | 4 | 40.5077 | -117.8585 |
| 114 | 2 | 39.3304 | -114.2145 |
| 131 | 9 | 39.1440 | -115.4363 |
| 132 | 6 | 38.3376 | -115.5150 |
| 133 | 2 | 37.9052 | -115.3496 |
| 134 | 3 | 38.3790 | -115.8939 |
| 161 | 8 | 38.9285 | -116.9361 |
| 162 | 1 | 38.7330 | -116.6541 |
| 163 | 4 | 38.6860 | -116.3187 |
| 173 | 5 | 38.7641 | -117.2749 |
| 181 | 8 | 39.4698 | -118.5795 |
| 182 | 3 | 39.7203 | -118.1836 |
| 183 | 4 | 39.7142 | -117.7866 |
| 184 | 6 | 39.3271 | -117.6816 |
| 202 | 6 | 38.5972 | -118.8369 |
| 205 | 7 | 38.7985 | -118.4336 |
| 206 | 2 | 38.2600 | -118.4121 |
| 208 | 1 | 38.1115 | -118.0947 |
| 211 | 8 | 37.8053 | -117.9742 |
| 212 | 18 | 37.6227 | -117.4266 |
| 213 | 7 | 38.2157 | -117.6918 |
| 223 | 8 | 37.8275 | -114.7799 |
| 241 | 9 | 37.3662 | -114.8642 |
| 243 | 7 | 37.0042 | -114.7089 |
| 244 | 8 | 36.6157 | -114.8116 |
| 245 | 6 | 37.4184 | -115.4631 |
| 252 | 2 | 37.1971 | -117.0256 |
| 253 | 16 | 36.7874 | -116.6464 |
| 254 | 5 | 36.6280 | -116.2055 |
| 261 | 5 | 36.4119 | -116.2209 |
| 262 | 16 | 36.1728 | -115.6276 |
| 263 | 16 | 35.6922 | -115.0971 |
| 264 | 5 | 35.2809 | -114.7325 |
| 266 | 13 | 35.8582 | -114.7987 |
| 268 | 17 | 36.3465 | -114.7850 |
| 269 | 10 | 36.0686 | -114.8374 |
| 271 | 10 | 36.9399 | -114.3471 |
| 272 | 6 | 36.3944 | -114.2025 |
| 280 | 3 | 36.7771 | -115.7715 |
| 281 | 4 | 36.8063 | -115.5533 |
| 282 | 2 | 36.7832 | -115.4103 |
| 284 | 2 | 36.6605 | -115.2379 |
| NTTR | 19 | 37.3149 | -116.3028 |

| **Table S3.** For the entropy analysis (Gompert et al., 2014) using only individuals from the four source herds (units 212, 268, 269, and 271), five separate models were run specifying the number of genetic clusters (*k*) from *k*=2 to *k*=4. For each *k*, the mean and standard devation (sd) of the deviance information criteria (DIC) are listed. | | |
| --- | --- | --- |
| *k* | Mean DIC | sd DIC |
| 2 | 3271820.2 | 80933.4 |
| 3 | 3743406.8 | 543208.2 |
| 4 | 3122372.0 | 50260.5 |

| **Table S4.** For the entropy analysis (Gompert et al., 2014) using all individuals, five separate models were run specifying the number of genetic clusters (*k*) from *k*=2 to *k*=7. For each *k*, the mean and standard devation (sd) of the deviance information criteria (DIC) are listed. | | |
| --- | --- | --- |
| *k* | Mean DIC | sd DIC |
| 2 | 19471724.9 | 430309.1 |
| 3 | 19400355.2 | 577057.7 |
| 4 | 19856269.8 | 982927.7 |
| 5 | 19733804.9 | 912000.3 |
| 6 | 19226106.5 | 737963.2 |
| 7 | 36971078.2 | 9393924.0 |

**Figure Legends**

**Fig. S1.** For each individual, an ancestry coefficient was estimated for each of two genetic ancestries (*k*) with entropy (Gompert et al., 2014). Ancestry coefficients from the *k* = 2 model with the lowest DIC are displayed.

**Fig. S2.** For each individual, an ancestry coefficient was estimated for each of three genetic ancestries (*k*) with entropy (Gompert et al., 2014). Ancestry coefficients from the *k* = 3 model with the lowest DIC are displayed.

**Fig. S3.** For each individual, an ancestry coefficient was estimated for each of five genetic ancestries (*k*) with entropy (Gompert et al., 2014). Ancestry coefficients from the *k* = 5 model with the lowest DIC are displayed.

**Fig. S4.** For each individual, an ancestry coefficient was estimated for each of six genetic ancestries (*k*) with entropy (Gompert et al., 2014). Ancestry coefficients from the *k* = 6 model with the lowest DIC are displayed.

**Fig. S5.** Principal component analysis (PCA) was used to document population genetic structure among desert bighorn sheep in Nevada. Panel A displays the relationship between principal components (PCs) one and two, while panel B depicts the relationship between PC3 and PC4. Hunt units within the same hunt management area are geographically proximate to one another (see Fig. 1) and are colored the same.

**Fig. S6.** Hudson’s *F*_ST_ (Hudson et al., 1992) was calculated based on allele frequencies for each pairwise combination of hunt units that each had at least five individuals genotyped. The histogram displays the distribution of *F*_ST_ across all pairwise combinations of hunt units, with bars colored to match those found in the heat map. The dashed horizontal line represents the mean pairwise *F*_ST_ for comparisons among the four remnant source herds (units 212, 268, 269, and 271).

**Fig. S7.** The relationship between haversine geographic distance and pairwise *F*_ST_ for (A) the subset of populations that have never received translocated individuals (*N* = 7) and (B) all populations. All included populations had at least five individuals genotyped.

**Fig. S8.** The relationship between the number of translocation sources and (A) expected heterozygosity or (B) observed heterozygosity is shown. See Table S1 for more information on translocation sources. All included populations had at least five individuals genotyped.

**Fig. S1**

**Fig. S2**

**Fig. S3**

**Fig. S4**

**Fig. S5**

**Fig. S6**

**Fig. S7**

**Fig. S8**

**Supplementary References**

Gompert, Z., Lucas, L. K., Buerkle, C. A., Forister, M. L., Fordyce, J. A., & Nice, C. C. (2014). Admixture and the organization of genetic diversity in a butterfly species complex revealed through common and rare genetic variants. *Molecular Ecology*, *23*, 4555-4573.

Hudson, R. R., Slatkin, M., & Maddison, W. P. (1992). Estimation of levels of gene flow from DNA sequence data. *Genetics*, *132*, 583-589.
